# Reactivity of 2-Ethoxyquinazolin- 4-yl hydrazine and its Use in Synthesis of Novel Quinazoline Derivatives of Antimicrobial Activity

**DOI:** 10.5539/gjhs.v4n1p174

**Published:** 2012-01-01

**Authors:** Maher A El-Hashash, Sameh A Rizk, Fakhry A El-Bassiouny, Khalid M Darwish

**Affiliations:** Faculty of Science, Chemistry Department Ain Shams University Abbassia, Cairo - Egypt; Science Faculty, Chemistry Department Garyounis University Benghazi-Libya Tel: 0020113049976 Email: khaliddarwish1962@yahoo.com

**Keywords:** Iminamine, Triazoloquinazoline, Sugar Hydrazones, *C*-Nucleosides

## Abstract

The reactions of 2-ethoxy-4-hydrazinoquinazoline 2 with diethyl oxalate and ethyl chloroacetate gave 6-ethoxy-2*H*-[1,2,4] triazino [4,3-*c*] quinazoline-3,4-dione 3 and 6-ethoxy-2,3-dihydro-4*H*-[1,2,4] triazino [4, 3-*c*] quinazolin-4-one 4 respectively. A series of 5-ethoxy-2-X-[1, 2, 4] triazolo [1, 5-c] quinazolines 5a-d was also produced by reacting 2 with the acid chlorides namely: benzoyl, crotonyl, cinnamyl and 2-furoyl chlorides via Dimroth rearrangement. Also, 2 reacted with ethyl chloroformate giving 6. Condensation of 2 with acetone gave Schiff base 7, and with monosaccharides gave the sugar hydrazones 8a-e which was thereafter acetylated giving the corresponding 9a-e. Cyclization of 8a-e by iron(III) chloride gave triazoloquinazolines 10a-e acyclic *C*-nucleosides which, by acetylation, afforded 11a-e. All products were confirmed by elemental, IR, MS, and ^1^H-NMR analysis. Products 8-11 were chosen for biological screening test against gram (+ ive) and gram (- ive) bacteria.

## 1. Introduction

Quinazolines are a big family of heterocyclic compounds, which have shown broad variety of biological activity profiles, e. g. analgesic, antiinflammatory, antipyretic [1, 2], antimicrobial [3], anticonvulsant [4], anticancer [5], antitumoral [6], antihypertensive [7], antimalarial [8], diuretic [9], antidiabetic [10], antihistamine/sedative [11], antibiotic [12] and many others. Heterocycle-bearing N-glycosides are well known to play a significant role as inhibitors. An example is the tetrazole-bearing N-glycosides used as SGLT2 inhibitors [13], where their hypoglycemic activity is tested *in vivo* by mice oral glucose tolerance test (OGTT). Moreover, sugar hydrazones exhibit remarkable biological activity [14]. Herein we report the synthesis of hydrazones of D-exoses and D-pentoses with 4-hydrazinoquinazoline and the screening of their antimicrobial potentials.

## 2. Result and Discussion

**Chart 1:**
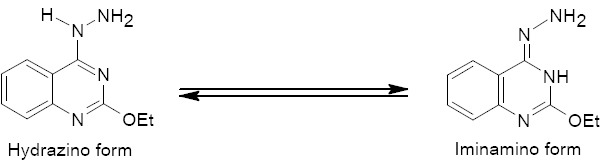
Tautomeric phenomenon of compound **2**

Recently, it was reported that 4-substituted-aminoquinazolines are exploited as potent antitumor compounds [15]. The 4-hydrazinoquinazolines resemble primary amines in being good substrates for aldehydes, ketones, alkyl and acid halides, anhydrides, etc. Therefore, they play a significant role in the synthesis of biologically active products [16]. The tautomeric behavior of hydrazinoquinazoline is used whenever necessary. For example, any necessary cyclization prior to product formation requires the presence of iminamine rather than hydrazine - form (Chart 1).

**Scheme 1:**
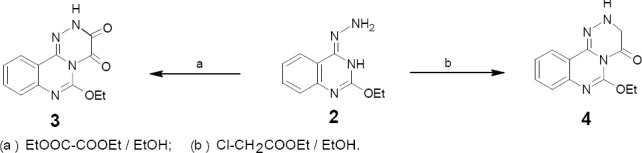
synthetic pathway for compounds **3** and **4**

Compound 2 reacted with diethyl oxalate and with ethyl chloroacetate in boiling ethanol to giving products 3 and 4 respectively (Scheme 1). The reaction possibly started with a nucleophilic attack of NH2 of hydrazine moiety on C=O of the ester group through a tetrahedral mechanism intermediate to yield a fleeting acyl derivative followed by 1,3-tautomerism and ring closure via SN2 mechanism.

**Scheme 2:**
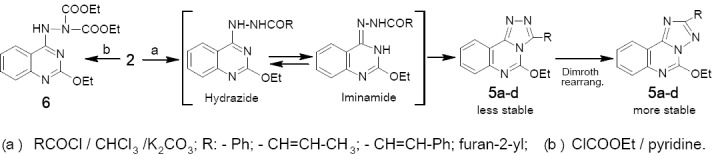
synthetic pathway for compounds **5a-d**

Similarly, compound 2 was reacted with acid halides namely: benzoyl, crotonyl, cinnamyl and furoyl chlorides in dry CHCl_3_ and K_2_CO_3_ giving the 2-acyl-1-(2-ethoxyquinazolin-4-yl)hydrazine derivatives, which tautomerized into the iminamide form upon heating and then underwent cyclization and Dimroth rearrangement affording the more stable derivatives 5a-d respectively (Scheme 2). Compound 2 was also reacted with ethyl chloroformate in dry pyridine affording derivative 6.

**Scheme 3:**
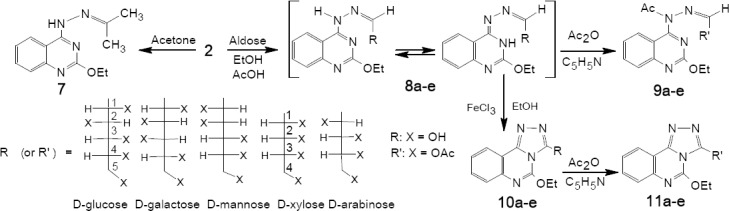
synthetic pathway for compounds **7 - 11**

Compound 2 was reacted with acetone affording derivative 7, whose mass spectrum showed a molecular ion peak at *m/z* 244,246 whereas the 1NMR spectrum showed a singlet at δ 2.40 ppm characteristic for CH_3_ groups of the hydrazone. A number of sugar hydrazones 8a-e were prepared by condensation of compound 2 with equimolar amounts of D-aldohexoses and D-aldopentoses namely: glucose, galactose, mannose, xylose and arabinose, respectively in boiling ethanol and drops of acetic acid as a catalyst (Scheme 3). Their IR spectra revealed characteristic absorption bands at 3459-3135 cm^-1^ attributed to OH and NH groups. Acetylation of these hydrazones 8a-e by acetic anhydride in pyridine at room temperature afforded the corresponding per-acetyl products 9a-e, whose IR spectra revealed disappearance of the bands of OH groups and appearance of absorption bands in the carbonyl group frequency region at 1711-1725 cm^-1^ and 1673 - 1692 cm^-1^ due to the OAc and NAc groups, respectively. The ^1^H-NMR spectra showed signals corresponding to *O*-acetyl groups in addition to NAc groups; whereas no signals could be found for NH groups confirming that per-*O*- and *N*-acetylation had taken place. The spectra also confirmed the presence of the HC=N proton as a doublet at δ 6.55-6.74 ppm low field in addition to the rest of alditol-1-yl side chain.

**Scheme 4:**
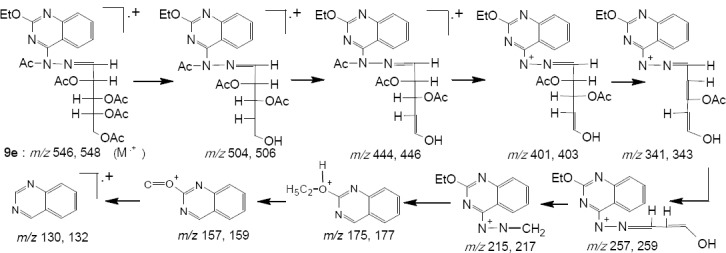
MS data interpretation of compound **9a**

The mass spectral data of 9e showed a molecular ion peak at *m/z* 546, 548 which agreed with the molecular formula C_25_H_30_N_4_O_10._ The ion at *m/z* 215, 217 confirmed a loss of sugar residue from the molecular ion. The fragment at *m/z* 175, 177 referred to quinazoline ring (Scheme 4).

**Scheme 5:**
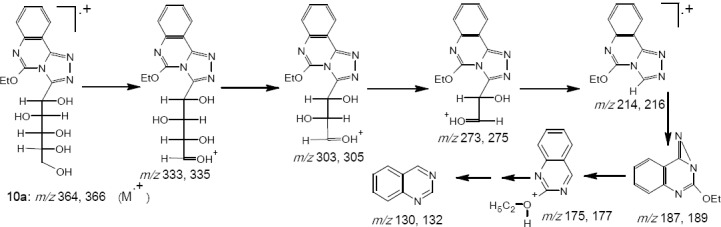
MS data interpretation of compound **10a**

The oxidative cyclization of the hydrazones 8a-e with ethanolic iron (III) chloride afforded the triazolo[4, 3-a]quinazolines 10a-e. The oxidation must have taken place by an electrophilic attack of the hard acid site of ferric chloride on the hardest basic site of sugar hydrazones 8a-e followed by an elimination of hydrogen chloride and formation of possibly a nitrilimine that undergoes 1,5-electrocyclization to give 10a-e. The IR spectra showed bands at 3240-3488 cm^-1^ (OH) and the mass spectral data of 10a showed a molecular ion peak at *m/z* 364 and 366 and an ion peak at *m/z* 214 and 216 presumably attributable to the triazoloquinazoline ring (Scheme 5).

**Scheme 6:**
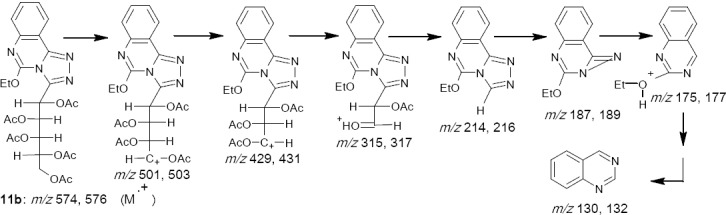
MS data interpretation of compound **11b**

The ^1^H-NMR spectrum of compound 10c showed a doublet at low field at δ 5.22 ppm assigned to H-1, followed by the rest of the alditol-1-yl chain at higher field. The spectrum of 10e is similar, showing a doublet at low field at δ 5.03 ppm for H-1. Acetylation of 10a-e by acetic anhydride in pyridine at room temperature gave polyacetoxyalkyl derivatives 11a-e, whose IR spectra showed only one absorption band in the C=O frequency region (OA). The OAc groups were confirmed by the ^1^H-NMR spectra showing singlets at δ 2.03-2.19. The doublets at δ 5.74-6.02 were attributed to H-1. The mass spectra of products 11b and 11d showed molecular ion peaks at *m/z* 574, 576 and 501, 503 (Scheme 6) which, on combination with the elemental analysis, led to the assignment of their molecular formulas C_26_H_30_N_4_O_11_ and C_23_H_26_N_4_O_9_ respectively. In addition, the characteristic fragment at *m/z* 214,216 was shown attributable to the triazoloquinazoline ring.

### 2.1 Antimicrobial Activity

All compounds were screened for their antimicrobial activity. Compounds 8-11 were tested against gram-positive bacteria *Staphylococcus aureus, Streptobacillus moniliformis* and *Bacillus subtillis* and gram-negative bacteria *E. coli, Streptobacillus moniliformis* and *Pseudomonas acru- ginosa* species applying the agar plate diffusion method. The screening results (Table 1) indicated that all the tested products exhibited antimicrobial activities against one or more type of bacteria. Almost all triazoloquinazoline products 10a-e and 11a-e showed more inhibition against the gram positive bacteria specially *Streptobacillus* than the gram negative one.

**Table 1:** Antimicrobial activity

Compd No	Gram-positive Bacteria			Gram-negative Bacteria		
	Staphylococcussp	Streptobacillussp	Bacillussubtillissp	Eschcolisp	Streptobacillusussp	Pseudomonassp
8a	**-**	**+**	**-**	**-**	**-**	**-**
8b	**-**	**+**	**-**	**-**	**-**	**-**
8c	**-**	**+**	**+**	**-**	**-**	**-**
8d	**-**	**+**	**-**	**-**	**-**	**-**
8e	**-**	**+**	**-**	**-**	**-**	**-**
9a	**-**	**+**	**+**	**-**	**+**	**-**
9b	**-**	**+**	**+**	**+**	**-**	**-**
9c	**-**	**+**	**+**	**-**	**-**	**+**
9d	**-**	**+**	**-**	**+**	**-**	**-**
9e	**-**	**+**	**+**	**+**	**-**	**+**
10a	**-**	**+**	**+**	**+**	**-**	**-**
10b	**-**	**+**	**+**	**+**	**+**	**+**
10c	**-**	**++**	**++**	**+**	**+**	**+**
10d	**+**	**+++**	**++**	**+**	**-**	**-**
10e	**+**	**+**	**+**	**+**	**-**	**-**
11a	**-**	**+++**	**++**	**+**	**+**	**+**
11b	**-**	**++**	**+**	**+**	**-**	**-**
11c	**-**	**++**	**+**	**+**	**-**	**-**
11d	**+**	**+**	**+**	**+**	**-**	**-**
11e	**-**	**++**	**+**	**+**	**-**	**-**

### 2.2 Experimental

All melting points recorded are uncorrected. The IR spectra were recorded on a Pye Unicam SP1200 spectrophotometer using KBr wafer technique. The ^1^H-NMR spectra were determined on a Varian FT-200 or Brucker AC-200 MHz instrument using TMS as an internal standard. Chemical shifts (δ) are expressed in ppm. The mass spectra were determined using MP model NS-5988 and Shimadzu single focusing mass spectrometer (70 eV). All the solvents used were of HPLC/AnalaR grade. All reagents were used as received from Alfa Aesar.

*Synthesis of 2-Ethoxyquinazolin-4-ylhydrazine 1*.

An emulsion of 4-chloro-2-ethoxyquinazoline 1 (0.01mol) and hydrazine hydrate (0.05 mol) in benzene (15 mL) was stirred for 2h. The benzene-insoluble gum obtained was treated and washed with water, dried and crystallized from ethanol affording reddish brown crystals of product 2. Evaporation of solvent from the benzene-soluble fraction afforded a residue which was rinsed with water and air dried. Crystallization of the residue from absolute ethanol afforded product 2.

*2-Ethoxyquinazolin-4-ylhydrazine* 2.

Yield 68%; m. p. 156-158°C. *Anal*. for C_10_H_12_N_4_O (M. wt. 204); Found: C, 58.86; H, 5.78; N, 27.45; Calcd: C, 58.82; H, 5.88; N, 27.45; IR υ (cm^-1^) 1620 (C=N), 3160 (NH), 3250, 3300 (NH_2_); MS: *m/z* [M+H]^+^ 204; ^1^H - NMR (DMSO-d_6_) δ 1.18 (t, 3H, CH3 of ethoxy *J* = 7.4 Hz), 4.19 (q, 2H, CH_2_ of ethoxy *J* = 7.4), 4.95 (br. s, 3H, NH and NH_2_), 7.43 - 8.08 (m, 4H, ArH).

*6-ethoxy-2H-[1,2,4]triazino[4,3-c]quinazoline-3,4-dione 3*.

A mixture of 2 (2.04 g, 0.01 mol) and diethyl oxalate (1.46 g, 0.01 mol) in boiling ethanol (30 mL) was heated under reflux for 10 h. After cooling the separated solid was collected and recrystallized from THF to give white crystals of 3; m. p. 237-239 ºC; yield 58 %. *Anal*. for C_12_H_10_N_4_O_3_ (M. wt. 258); Found: C, 54.21; H, 3.56; N, 21.89; Calcd: C, 55.81; H, 3.88; N, 21.71; IR υ (cm^-1^) 1680 -1690 (C=O), 3275 (sec NH); MS: *m/z* [M+H]^+^ 258 (77%). ^1^H-NMR (DMSO-d_6_) δ 1.21 (t, 3H, CH_3_ of ethoxy *J* = 7.4 Hz), 4.38 (q, 2H, CH_2_ of ethoxy *J* = 7.4), 7.21 -8.13(m, 5H, ArH nd NH), 10.20(s, 1H, NH, exchangeable).

*6-ethoxy-2H-[1,2,4]triazino[4,3-c]quinazolin-3(4H)-one 4*.

A mixture of 2 (2.04 g, 0.01 mol) and ethyl chloroacetate (1.22 g, 0.01 mol) in boiling ethanol (35 mL) was heated under reflux for 10 h. The solid that separated after cooling was recrystallized from dioxane affording light brown crystals of 4; m. p. 213-216 ºC; yield 58 %. *Anal*. for C_12_H_12_N_4_O_2_ (M. wt. 244); Found: C, 58.85; H, 4.53; N, 23.02; Calcd: C, 59.02; H, 4.92; N, 22.95; IR υ (cm^-1^) 1675 (C=O), 2993 (CH), 3312 (sec NH); MS: *m/z* [M+H]^+^ 244; ^1^H-NMR (DMSO-d_6_) δ 1.19 (t, 3H, CH_3_ of ethoxy *J* = 7.4 Hz), 3.94, 4.30(m, 2H, CH_2_CO), 4.30 (q, 2H, CH_2_ of ethoxy *J* = 7.4), 7.01- 8.31 (m, 5H, ArH and NH of triazine), 9.96 (s, 1H, NH, exchangeable).

*Synthesis of 5-ethoxy-2-substituted[1,2,4]triazolo[1,5-c]quinazoline 5a-d*.

To a solution of derivative 2 (0.01mol) in dry chloroform (100 mL) containing anhydrous K_2_CO_3_ (1g) the acid chloride namely: benzoyl, crotonyl, cinnamyl and furoyl chlorides (0.015 mol) was added slowly. After the addition was complete, the mixture was stirred at room temperature for 30 min and then heated on a steam bath for1h. The mixture was filtered, evaporated and the crude product was collected and crystallized from the proper solvent affording product 5a-d.

*5-Ethoxy-2-phenyl[1,2,4]triazolo[1,5-c]quinazoline 5a*.

Colorless needles from ethanol; m. p. 168 -170ºC; yield 68%. *Anal*. for C_17_H_14_N_4_O (M. wt. 290); Found: C, 70.17; H, 4.91; N, 19.38; Calcd: C, 70.34; H, 4.83; N, 19.31; IR υ (cm^-1^) 1622 (C=N), 3050 (CH); MS: *m/z* [M+H]^+^ 290 (32.2), 292 (12.3), 214 (100), 216 (23.5), 174 (43.8), 176 (8.2), 78 (13.2), 80 (0.3); ^1^H-NMR (DMSO-d_6_) δ 1.19 (t, 3H, CH_3_ of ethoxy *J* = 7.4), 4.33 (q, 2H, CH_2_ of ethoxy *J* = 7.4), 7.57-8.10 (m, 5H, phenyl), 7.62 - 8.65 (m, 4H, ArH).

*5-Ethoxy-2-[(1E)-prop-1-en-1-yl][1,2,4]triazolo[1,5-c]quinazoline* 5b.

Brown white crystals from ethanol; m. p. 223-225 ºC; yield 71%. *Anal*. for C_14_H_14_N_4_O (M. wt. 254); Found: C, 66.28; H, 5.31; N, 22.23; Calcd: C, 66.14; H, 5.51; N, 22.05; IR υ (cm^-1^) 1635 (C=N), 3050 (CH); MS: *m/z* [M+H]^+^ 254 (48.2), 256 (14.2), 174 (100), 176 (38.1); ^1^H-NMR(DMSO-d_6_) δ 1.21(t, 3H, CH_3_ of ethoxy *J* = 7.4), 1.67 (t, 3H, CH_3_), 4.31(q, 2H, CH_2_ of ethoxy *J* = 7.4 Hz), 6.13 (d, H, CH_trans_), 6.70 (d, H, CH_trans_), 7.53 - 8.21 (m, 4H, ArH).

*5-Ethoxy-2-[(E)-2-phenylethenyl][1,2,4]triazolo[1,5-c]quinazoline 5c*.

Off-white crystals from ethanol; 153 - 155 ºC; yield 62%. *Anal*. for C_19_H_16_N_4_O (M. wt. 316); Found: C, 72.84; H, 5.19; N, 17.76; Calcd: C, 72.15; H, 5.06; N, 17.72; IR υ (cm^-1^) 1633(C=N), MS: m/z [M+H]^+^ 316 (29.3), 318 (12.8), 174 (100), 176 (41.1), 103 (12.7), 105 (0.8); ^1^H-NMR(DMSO-d_6_) δ 1.2(t, 3H, CH_3_ of ethoxy *J* = 7.4 Hz), 4.38 (q, 2H, CH_2_ of ethoxy *J* = 7.4), 7.09, 7.48 (2d, 2H, of two olefin protons), 7.4-7.6 (m, 5H, PhH), 7.67-8.71 (m, 4H, quinazoline).

*5-Ethoxy-2-(furan-2-yl)[1,2,4]triazolo[1,5-c]quinazoline 5d*.

White crystals from benzene; 163-164ºC; yield 68%. *Anal*. for C_15_H_12_N_4_O_2_ (M. wt. 280); Found: C, 64.38; H, 4.31; N, 20.07; Calcd: C, 64.29; H, 4.29; N, 20.00; IR υ (cm^-1^) 1619 (C=N), MS: *m/z* [M+H]^+^ 280 (33.2), 282 (12.4), 174 (100), 176 (31.3), 60 (0.8), 61 (0.1); ^1^H-NMR(DMSO-d_6_) δ1.2 (t, 3H, CH_3_ of ethoxy *J* = 7.4Hz), 4.2 (q, 2H, CH_2_ of ethoxy *J* = 7.4), 6.79(dd, 1H, *J* = 3.6Hz, *J* = 1.6, Furan-H), 7.29(d, 1H, *J* = 4.4Hz, Furan-H), 7.76(d, 1H, *J* = 1.6Hz, Furan-H), 7.5 - 8.2 (m, 4H, ArH).

*1-(2-Ethoxyquinazolin-4-yl)-2-bis(ethoxycarbonyl) hydrazine* 6.

A mixture of 2 (0.01 mol) and ethyl chloroformate (0.02 mol) in dry pyridine (20 mL) was heated at boiling water bath for 4 h. The solvent was evaporated under vacuum, the residue was cooled and crystallized from ethanol giving colorless needles of 6; m.p. 123-125 ºC; yield 52 %. *Anal*. C_16_H_20_N_4_O_5_ (M.wt. 348); Found: C, 55.27; H, 5.83; N, 16.08; Calcd: C, 55.17; H, 5.75; N, 16.09; IR υ (cm^-1^) 1250 (C-O), 1622 (C=N), 1731 (C=O), 2986 (C-H); MS: *m/z* [M+H]^+^ 348 (31.5), 350 (14.1), 275 (2.8), 277 (13.2), 187 (0.8), 189 (0.2), 174 (100), 176 (39.4), 74 (0.7), 75(0.1); ^1^H-NMR(DMSO-d_6_) δ 1.11-1.25 (t, 9H, 3 CH_3_ of ethoxy), 4.15-4.25(q, 6H, 3 CH_2_ of ethoxy), 8.5-8.8(m, 4H, ArH), 10.05(s, 1H,NH).

*2-ethoxy-4-hydrazinoquinazoline Acetone hydrazone 7*.

A solution of crude 2 (0.01 mol) in acetone was left to stand for several days when the solvent had evaporated to give a solid from which the hydrazone 7 (80%) was isolated by chromatography on silica gel (30 g, 2.5% absolute ethanol-chloroform). Crystallization from hexane gave product 7 as colorless solid that turned deep yellow on exposure to light and air; m. p. 115 -116 ºC; yield 85 %. *Anal*. for C_13_H_16_N_4_O (M. wt. 244); Found: C, 63.98; H, 6.61; N, 22.95; Calcd: C, 63.93; H, 6.56; N, 22.95; IR υ (cm^-1^) 1634 (C=N), 2993 (CH), 3243 (sec NH); MS: *m/z* [M+H]^+^ 244 (33.1), 246 (12.6), 215 (100), 217 (19.6), 174 (55.8), 176 (1.2); ^1^H-NMR(DMSO-d_6_) δ 1.13 (t, 3H, CH_3_ of ethoxy *J* = 7.4), 2.4 (3H, s, -N=C-CH_3_), 4.23(q, 2H, CH_2_ of ethoxy *J* = 7.4), 7.1-8.3(m, 4H, ArH), 8.3(br. s,1H, NH).

*General procedure for the synthesis of sugar (2-ethoxyquinazolin-4-yl) hydrazones* 8a-e.

To a suspension of 2-Ethoxy-4-hydrazinoquinazoline 2 (0.01 mol) in ethanol (30 ml), was added a solution of selected sugar (D-glucose, D-galactose, D-mannose, D-xylose and D-arabinose (0.01 mol)) in water (10 ml) and few drops of glacial acetic acid. The mixture was heated under reflux until reaction was judged complete by TLC (2-6 h). The solid product formed upon cooling was filtered off, washed with the minimum amount of ethanol, dried and finally crystallized from ethanol to afford the corresponding hydrazones 8a-e.

*2-ethoxy-4-hydrazinoquinazoline-D-glucose hydrazone* 8a.

Yield 62 % (from ethanol); m. p. 212 - 214 ºC; *Anal*. for C_16_H_22_N_4_O_6_ (M. wt. 366); Found: C, 52.66; H, 6.16; N, 15.41; Calcd: C, 52.46; H, 6.01; N, 15.30; IR υ (cm^-1^) 1615 (C=N), 3225 - 3417 (OH, NH); MS: *m/z* [M+H]^+^ 366; ^1^H-NMR (DMSO-d_6_) δ 1.17 (t, 3H, CH_3_ of ethoxy *J* = 7.4), 4.17 (q, 2H, CH_2_ of ethoxy *J* = 7.4), 7.1- 8.3 (m, 4H, ArH), 8.33 (br. s, 1H, NH).

*2-ethoxy-4-hydrazinoquinazoline-D-galactose hydrazone* 8b.

Yield 88% (from DMF/ethanol); m. p. 193-195 ºC; *Anal*. for C_16_H_22_N_4_O_6_ (M. wt. 366); Found: C, 52.56; H, 6.23; N, 15.48; Calcd: C, 52.46; H, 6.01; N, 15.30; IR υ (cm^-1^) 1615 (C=N), 3135-3391 (OH and NH); MS: *m/z* [M+H]^+^ 366; ^1^H-NMR (DMSO-d_6_) δ 1.13 (t, 3H, CH_3_ of ethoxy *J* = 7.4 Hz), 4.21 (q, 2H, CH_2_ of ethoxy *J* = 7.4), 7.3 - 8.2 (m, 4H, ArH), 8.37 (br. s, 1H, NH).

*2-ethoxy-4-hydrazinoquinazoline-D-mannose hydrazone* 8c.

Yield 77% (from DMF/ethanol); m. p. 222-224ºC; *Anal*. for C_16_H_22_N_4_O_6_ (M. wt. 366); Found: C, 52.42; H, 6.04; N, 15.37; Calcd: C, 52.46; H, 6.01; N, 15.30; IR υ (cm^-1^) 1618 (C=N), 3232-3459 (OH and NH); MS: *m/z* [M+H]^+^ 366; ^1^H-NMR (DMSO-d_6_) δ 1.15 (t, 3H, CH_3_ of ethoxy *J* = 7.4 Hz), 4.23 (q, 2H, CH_2_ of ethoxy *J* = 7.4), 7.0 - 8.1 (m, 4H, ArH), 8.29 (br. s, 1H, NH).

*2-ethoxy-4-hydrazinoquinazoline-D-ribose hydrazone* 8d.

Yield 61% (from DMF/ethanol); m. p. 217-219 ºC; *Anal*. for C_15_H_20_N_4_O_5_ (M. wt. 336); Found: C, 53.68; H, 6.04; N, 16.57; Calcd: C, 53.57; H, 5.95; N, 16.67; IR υ (cm^-1^) 1616 (C=N), 3210-3439 (OH and NH); MS: *m/z* [M+H]^+^ 336; ^1^H-NMR (DMSO-d_6_) δ 1.13 (t, 3H, CH3 of ethoxy *J* = 7.4 Hz), 4.23 (q, 2H, CH_2_ of ethoxy *J* = 7.4), 7.1- 8.2 (m, 4H, ArH), 8.52 (br. s, 1H, NH).

*2-ethoxy-4-hydrazinoquinazoline-D-arabinose hydrazone* 8e.

Yield 63% (from ethanol); m. p. 197-198 ºC; *Anal*. for C_15_H_20_N_4_O_5_ (M. wt. 336); Found: C, 53.62; H, 5.98; N, 16.63; Calcd: C, 53.57; H, 5.95; N, 16.67; IR υ (cm^-1^) 1613 (C=N), 3230-3414 (OH and NH); MS: *m/z* [M+H]^+^ 336; ^1^H-NMR (DMSO-d_6_) δ 1.11 (t, 3H, CH_3_ of ethoxy *J* = 7.4 Hz), 4.19 (q, 2H, CH_2_ of ethoxy *J* = 7.4), 7.2- 8.3 (m, 4H, ArH), 8.33 (br. s, 1H, NH).

*Synthesis of per-O-acetylsugar [1-acetyl-1-(2-ethoxyquinazolin-4-yl)] hydrazones* 9a-e.

A cold solution of 8a-e (0.02 mol) in dry pyridine (50 mL) was treated with Ac_2_O (50 mL). The mixture was kept overnight at room temperature, with occasional shaking, and then poured onto crushed ice, and the residue was collected by filtration, washed repeatedly with water, dried and recrystallized from ethanol affording product 9a-e.

*2,3,4,5,6-Penta-O-acetyl-D-glucose[1-acetyl-1-(2-ethoxyquinazolin-4-yl)] hydrazones* 9a.

Yield 58 %; m. p. 63 - 64 ºC; *Anal*. for C_28_H_34_N_4_O_12_ (M. wt. 618); Found: C, 54.41; H, 5.56; N, 9.01; Calcd: C, 54.37; H, 5.50; N, 9.06; IR υ (cm^-1^) 1608 (C=N), 1673 (NAc), 1718 (OAc); MS: *m/z* [M+H]^+^ 618; ^1^H-NMR (DMSO-d_6_) δ 1.13 (t, 3H, CH_3_ of ethoxy *J* = 7.4 Hz), 2.02, 2.04, 2.10 (3s, 15H, 5 OAc), 2.50 (s, 3H, NAc), 4.1(q, 2H, CH_2_ of ethoxy *J* = 7.4),4.15 (dd, 1H, H-6’), 4.26 (dd, 1H, H-6), 5.02-5.10 (m, 1H, H-5), 5.44-5.55 (m, 2H, H-4, H-3), 5.62 (dd, 1H, H-2), 6.74 (d, 1H, H-1), 7.11-8.23 (m, 4H, ArH).

*2,3,4,5,6-Penta-O-acetyl-D-galactose[1-acetyl-1-(2-ethoxyquinazolin-4-yl)] hydrazones* 9b.

Yield 80 %; m. p. 158 -160 ºC; *Anal*. for C_28_H_34_N_4_O_12_ (M. wt. 618); Found: C, 54.43; H, 5.53; N, 9.03; Calcd: C, 54.37; H, 5.50; N, 9.06; IR υ (cm^-1^) 1633(C=N), 1692(NAc), 1722(OAc); MS: *m/z* [M+H]^+^ 618; ^1^H-NMR (DMSO-d_6_) δ 1.13 (t, 3H, CH_3_ of ethoxy *J* = 7.4 Hz), 1.96, 1.99, 2.02, 2.03, 2.08, 2.09 (5s, 15H, 5OAc), 2.47(s, 3H, NAc), 3.88(dd, 1H, H-6’), 4.1(q, 2H, CH_2_ of ethoxy *J* = 7.4), 4.28 (dd, 1H, H-6), 5.38-5.88 (m, 4H, H-5, H-4, H-3, H-2), 6.55 (d, 1H, H-1), 7.15- 8.33 (m, 4H, ArH).

*2,3,4,5,6-Penta-O-acetyl-D-mannose[1-acetyl-1-(2-ethoxyquinazolin-4-yl)] hydrazones* 9c.

Yield 63 %; m. p. 58 - 60 ºC; *Anal*. for C_28_H_34_N_4_O_12_ (M. wt. 618); Found: C, 54.40; H, 5.51; N, 9.02; Calcd: C, 54.37; H, 5.50; N, 9.06; IR υ (cm^-1^) 1615(C=N), 1682(NAc), 1711(OAc); MS: *m/z* [M+H]^+^ 618; ^1^H-NMR (DMSO-d_6_) δ 1.13 (t, 3H, CH_3_ of ethoxy *J* = 7.4 Hz), 2.05, 2.06, 2.10 (3s, 15H,5OAc), 2.52 (s, 3H, NAc), 4.11(q, 2H, CH_2_ of ethoxy *J* = 7.4), 4.14 (dd, 1H, H-6’), 4.28 (dd, 1H, H-6), 5.22-5.40 (m, 1H, H-5), 5.42 (d, 1H, H-4), 5.54 (dd, 1H, H-3), 5.66 (dd, 1H, H-2), 6.68 (d, 1H, H-1), 7.00 - 8.13 (m, 4H, ArH).

*2,3,4,5-Tetra-O-acetyl-D-ribose[1-acetyl-1-(2-ethoxyquinazolin-4-yl)] hydrazones* 9d.

Yield 52 %; m. p. 92 - 93 ºC; *Anal*. for C_25_H_30_N_4_O_10_ (M. wt. 546); Found: C, 54.97; H, 5.54; N, 10.29; Calcd: C, 54.94; H, 5.49; N, 10.26; IR υ (cm^-1^) 1619 (C=N), 1682 (NAc), 1725 (OAc); MS: *m/z* [M+H]^+^ 546; ^1^H-NMR (DMSO-d_6_) δ 1.91, 1.94, 2.00, 2.19 (4s, 12H, 4OAc), 1.13 (t,3H, CH_3_ of ethoxy *J* = 7.4 Hz), 2.51 (s, 3H, NAc), 4.15 (q, 2H, CH_2_ of ethoxy *J* = 7.4), 4.22 (dd, 1H, H-5’), 4.39 (dd, 1H, H-5), 5.38 - 5.40 (m, 1H, H-4), 5.78 (dd, 1H, H-3), 6.01 (dd, 1H, H-2), 6.59 (d, 1H, H-1), 7.10- 8.32 (m, 4H, ArH).

*2,3,4,5-Tetra-O-acetyl-D-arabinose[1-acetyl-1-(2-ethoxyquinazolin-4-yl)] hydrazones* 9e.

Yield 67 %; m. p. 108-111 ºC; *Anal*. for C_25_H_30_N_4_O_10_ (M. wt. 546); Found: C, 54.95; H, 5.52; N, 10.27; Calcd: C, 54.94; H, 5.49; N, 10.26; IR υ (cm^-1^) 1610 (C=N), 1682 (NAc), 1715 (OAc); MS: *m/z* [M+H]^+^ 546 (42.9), 548 (14.3), 504 (10.5), 506 (3.4), 444 (25.0), 446 (8.2), 401 (13.3), 403 (4.4), 341 (17.6), 343 (5.8), 257 (40.5), 259 (13.4), 215 (100), 217 (33.3), 175 (16.2), 177 (5.3), 157 (2.8), 159 (0.9), 130 (58.3), 132 (0.1).

*General method for preparing 1-(alditol-1-yl) - 5-ethoxy[1,2,4]triazolo[1,5-c] quinazoline* 10a-e.

A 2 M solution of iron (III) chloride in EtOH (2 mL) was added dropwise to a boiling solution of 8a-e (0.01 mol) in ethanol (50 mL). Heating was continued for 10 min and the mixture was then kept overnight at room temperature. The product was filtered, washed repeatedly with water, air dried and recrystallized from EtOH affording product 10a-e.

*1-(D-gluco-pentitol-1-yl)-5-ethoxy[1,2,4]triazolo[1,5-c] quinazoline* 10a.

Yield 90 %; m. p. 85-87 ºC; *Anal*. for C_16_H_20_N_4_O_6_ (M. wt. 364); Found: C, 52.79; H, 5.52; N,15.43; Calcd: C, 52.75; H, 5.49; N, 15.38; IR υ (cm^-1^) 1613 (C=N), 3240-3454 (OH); MS: *m/z* [M+H]^+^ 364 (14.4), 366 (4.8), 333 (0.3), 335 (0.1), 303 (0.6), 305 (0.2), 273 (0.6), 275 (0.2), 214 (100), 216 (33.4), 187 (59.8), 189 (19.6), 175(6.2), 177 (2.1), 157 (2.5), 159 (0.5), 130(63.4), 132 (0.1).

*1-(D-galacto-pentitol-1-yl)-5-ethoxy[1,2,4]triazolo[1,5-c] quinazoline* 10b.

Yield 85 %; m. p. 80 ºC; *Anal*. for C_16_H_20_N_4_O_6_ (M. wt. 364); Found: C, 52.77; H, 5.56;N, 15.48; Calcd: C, 52.74; H, 5.49; N, 15.38; IR υ (cm^-1^) 1619 (C=N), 3340-3450 (OH);

*1-(D-manno-pentitol-1-yl)-5-ethoxy[1,2,4]triazolo[1,5-c] quinazoline* 10c.

Yield 75 %; m. p. 101-102ºC; *Anal*. for C_16_H_20_N_4_O_6_ (M. wt. 364); Found: C, 52.82; H, 5.59; N, 15.53; Calcd: C, 52.74; H, 5.49; N, 15.38; IR υ (cm^-1^) 1615 (C=N), 3290-3466 (OH); ^1^H-NMR (DMSO-d_6_) δ 3.97-4.20 (m, 2H, H-5’, H-5), 4.40 (dd, 1H, H-4), 4.62 (dd, 1H, H-3), 5.03 (t, 1H, H-2), 5.22 (d, 1H, H-1), 7.10- 8.32 (m, 4H, ArH).

*1-(D-ribo-pentitol-1-yl)-5-ethoxy[1,2,4]triazolo[1,5-c] quinazoline* 10d.

Yield 68 %; m. p. 128 ºC; *Anal*. for C_15_H_18_N_4_O_5_ (M. wt. 334); Found: C, 53.92; H, 5.43; N, 16.81; Calcd: C, 53.89; H, 5.39; N, 16.77; IR υ (cm^-1^) 1623 (C=N), 3310 - 3444 (OH); MS: *m/z* [M+H]^+^ 334 (21.1), 336 (7.0), 303 (3.5), 305 (1.1), 273 (0.6), 275 (0.2), 214 (9.9), 216 (3.2), 187 (100), 189 (33.2), 175 (5.4), 177 (1.1), 157 (1.8), 159 (0.3), 130(56.4), 132 (0.1).

*1-(D-arabino-pentitol-1-yl)- 5-ethoxy[1,2,4]triazolo[1,5-c] quinazoline* 10e.

Yield 63 %; m. p. 108 ºC; *Anal*. for C_15_H_18_N_4_O_5_ (M. wt. 334); Found: C, 53.97; H, 5.46; N, 16.88; Calcd: C, 53.89; H, 5.39; N, 16.77; IR υ (cm^-1^) 1619 (C=N), 3320-3480 (OH); ^1^H-NMR (DMSO-d_6_) δ 3.49-3.75 (m, 2H, H-4’, H-4), 4.18-4.39 (m, 2H, H-3, H-2), 5.03 (d, 1H, H-1),7.1-8.3 (m, 4H, ArH).

*Preparation of 1-(penta-O-acetylsugar-1-yl)-5-ethoxy-1,2,4-triazolo[1,5-c] quinazoline* 11a-e

A cold solution of 10a-e (0.002 mol) in dry pyridine (10 mL) was treated with Ac_2_O (6 mL), and the mixture was kept overnight at room temperature, with occasional shaking, and then poured onto crushed ice, and the residue was collected by filtration, washed repeatedly with water, dried and recrystallized from ethanol affording product 11a-e.

*1-(1,2,3,4,5-penta-O-acetyl-D-glucopentitol-1-yl)-5-ethoxy-1,2,4-triazolo[1,5-c] quinazoline* 11a.

Yield 78 %; m. p. 71 ºC; *Anal*. for C_26_H_30_N_4_O_11_ (M. wt. 574); Found: C, 54.42; H, 5.26; N, 9.83; Calcd: C, 54.36; H, 5.23; N, 9.76; IR υ (cm^-1^) 1650 (C=N), 1725 (OAc); ^1^H-NMR (DMSO-d_6_) δ 1.99, 2.01, 2.03 (3s, 12H, 4OAc), 3.98 (dd, 1H, H-5’), 4.30 (dd, 1H, H-5), 5.30-5.52 (m, 3H, H-4, H-3, H-2), 5.86 (d, 1H, H-1), 7.26-8.30 (m, 4H, ArH).

*1-(1,2,3,4,5-penta-O-acetyl-D-galactopentitol-1-yl)-5-ethoxy-1,2,4-triazolo[1,5-c] quinazoline* 11b.

Yield 81 %; m. p. 66 ºC; *Anal*. for C_26_H_30_N_4_O_11_ (M. wt. 574); Found: C, 54.39; H, 5.24; N, 9.79; Calcd: C, 54.36; H, 5.23; N, 9.76; IR υ (cm^-1^) 1619(C=N), 1719(OAc); MS:*m/z* [M+H]^+^ 574 (19.3), 576 (6.3), 501 (27.4), 503(9.1), 429(31.7), 431 (10.5), 315 (9.5), 317 (3.2), 214 (100), 216 (33.3), 187 (59.8), 189 (19.9), 175(5.4), 177 (2.1), 157 (1.3), 159 (0.2), 130 (48.4), 132 (0.1).

*1-(1,2,3,4,5-penta-O-acetyl-D-mannopentitol-1-yl)-5-ethoxy-1,2,4-triazolo[1,5-c]quinazoline* 11c.

Yield 63 %; m. p. 76 ºC; *Anal*. for C_26_H_30_N_4_O_11_ (M. wt. 574); Found: C, 54.41; H, 5.27;N, 9.81; Calcd: C, 54.36; H, 5.23; N, 9.76; IR υ (cm^-1^) 1639 (C=N), 1745 (OAc); ^1^H-NMR (DMSO-d_6_) δ1.96, 1.99, 2.03, 2.08(4s, 12H, 4OAc), 3.90(dd, 1H, H-5’), 4.32 (dd, 1H, H-5), 5.30-5.53 (m, 3H, H-4, H-3, H-2), 5.76 (d, 1H, H-1), 7.15-8.31(m, 4H, ArH).

*1-(1,2,3,4-tetra-O-acetyl-D-ribopentitol-1-yl)-5-ethoxy-1,2,4-triazolo[1,5-c] quinazoline* 11d.

Yield 58 %; m. p. 71 ºC; *Anal*. for C_23_H_26_N_4_O_9_ (M. wt. 502); Found: C, 54.94; H, 5.22; N, 11.14; Calcd: C, 54.98; H, 5.18; N, 11.16; IR υ (cm^-1^) 1629 (C=N), 1731 (C=O); MS: *m/z* [M+H]^+^ 502 (21.3), 504 (7.1), 430 (17.6), 432 (5.8), 358 (21.4), 360 (7.1), 286(16.9), 288(5.6), 214 (100), 216 (33.2), 187(8.4), 189(2.8), 175(5.8), 177(1.9), 157(1.6), 159 (0.1), 130 (59.3), 132 (0.1). ^1^H-NMR (DMSO-d_6_) δ 1.13 (t, 3H, CH_3_ of ethoxy J = 7.4), 2.03, 2.06, 2.08 (3s, 12H, 4OAc), 4.05 (dd, 2H, H-4’, H-4), 4.35-4.55 (m, 1H, H-3), 5.56(dd, 1H, H-2), 5.74 (d, 1H, H-1), 7.1 - 8.3 (m, 4H, ArH).

*1-(1,2,3,4-tetra-O-acetyl-D-arabinopentitol-1-yl)-5-ethoxy-1,2,4-triazolo[1,5-c] quinazoline* 11e. Yield 67%; m. p. 98ºC; *Anal*. for C_23_H_26_N_4_O_9_ (M. wt. 502); Found: C, 54.92; H, 5.24; N, 11.19; Calcd: C, 54.98; H, 5.18; N, 11.16; IR υ (cm^-1^) 1618 (C=N); ^1^H-NMR (DMSO-d_6_) δ 1.13 (t, 3H, CH_3_ of ethoxy J = 7.4), 1.81, 1.86, 1.91, 2.19 (4s, 12H, 4 OAc), 4.22 (dd, 2H, H-4’, H-4), 5.35 - 5.41 (m, 1H,H-3), 5.76 (dd, 1H, H-2), 6.02 (d, 1H, H-1), 7.17 - 8.32 (m, 4H, ArH).
